# Can We Deliver Person-Centred Obesity Care Across the Globe?

**DOI:** 10.1007/s13679-022-00489-7

**Published:** 2022-10-22

**Authors:** Louisa J. Ells, Mark Ashton, Rui Li, Jennifer Logue, Claire Griffiths, Gabriel Torbahn, Jordan Marwood, James Stubbs, Ken Clare, Paul J. Gately, Denise Campbell-Scherer

**Affiliations:** 1 Obesity Institute, School of Health, Leeds Beckett University, Leeds, UK; 2 Huddersfield Road Surgery, Sheffield, UK; 3 Lancaster Medical School, Lancaster University, Lancaster, UK; 4 Obesity Institute, Carnegie School of Sport, Leeds Beckett University, Leeds, UK; 5 Department of Pediatrics, Paracelsus Medical University, Klinikum Nürnberg, Universitätsklinik Der Paracelsus Medizinischen Privatuniversität Nürnberg, Nuremberg, Germany; 6 School of Psychology, University of Leeds, Leeds, UK; 7 Obesity UK, Leeds UK; 8 Obesity Institute, Carnegie School of Sport, Leeds Beckett University, Leeds, UK; 9 MoreLife UK Ltd, Leeds, UK; 10 Physician Learning Program, Faculty of Medicine & Dentistry, University of Alberta, Edmonton, Canada; 11 Department of Family Medicine, Faculty of Medicine & Dentistry, University of Alberta, Edmonton, Canada

**Keywords:** Obesity, Person-centred care, Healthcare systems, Complex systems

## Abstract

**Purpose of Review:**

This article discusses what person-centred care is; why it is critically important in providing effective care of a chronic, complex disease like obesity; and what can be learnt from international best practice to inform global implementation.

**Recent Findings:**

There are four key principles to providing person-centred obesity care: providing care that is coordinated, personalised, enabling and delivered with dignity, compassion and respect. The Canadian 5AsT framework provides a co-developed person-centred obesity care approach that addresses complexity and is being tested internationally.

**Summary:**

Embedding person-centred obesity care across the globe will require a complex system approach to provide a framework for healthcare system redesign, advances in people-driven discovery and advocacy for policy change. Additional training, tools and resources are required to support local implementation, delivery and evaluation. Delivering high-quality, effective person-centred care across the globe will be critical in addressing the current obesity epidemic.

**Graphical Abstract:**

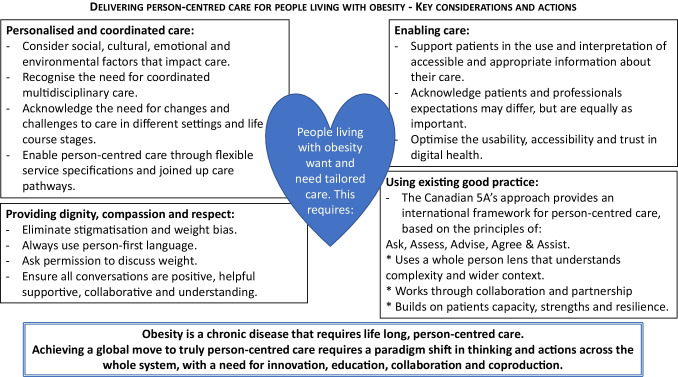

## Introduction

Obesity is a chronic disease defined by dysfunctional or excessive adiposity which impairs health [1, 2]. The prevalence of obesity has now reached epidemic proportions with over 650 million adults and 340 million children and adolescents affected globally [3]. Although once considered a disease of high-income countries, obesity prevalence is now increasing in middle- and low-income countries, with some countries now facing a double burden of over- and under-nutrition [4]. It is also important to understand that obesity does not affect all groups equally with significant socio-economic, gender and ethnic inequalities [5], which have further widened as a result of the COVID-19 pandemic [6].

The medical complications associated with obesity can impact almost every body system, resulting in serious life-threatening and limiting diseases such as type 2 diabetes, cardiovascular disease, several cancers and a number of respiratory, reproductive, psychological and muscular-skeletal conditions [7]. Obesity therefore significantly impacts people, families, healthcare systems, society and the wider economy. Current estimates suggest that excess weight costs global health services US$990 billion per year (13% of total healthcare expenditure) [8]. As a result, effectively managing obesity remains a global priority, yet despite significant research and service investment, the battle in addressing this epidemic remains far from over.

The challenge to successful management is the complex relapsing nature of obesity, driven by over hundred interlinked determinants, spanning biology, psychology, society and the food and activity environments [9]. This is in addition to the syndemic effects of the socio-cultural context of vulnerable populations [10]. The rapid changes to the environment have undoubtedly influenced the recent increases in obesity prevalence observed over the last three decades [3], with hundreds of gene polymorphisms interacting with the obesogenic environment to influence body weight [11]. It is perhaps therefore unsurprising that the short-term “one-size-fits-all” management strategy is failing, with an urgent need to provide more holistic person-centred, long-term obesity care, which embraces the complexity of treatment [12].

## What is Person-centred Care?

In the absence of a globally recognised definition, the Health Foundation describe person-centred care as supporting people to “develop the knowledge, skills and confidence they need to more effectively manage and make informed decisions about their own health and health care” [13]. Whilst person-centred care is not a new concept for countries such as Canada [14], for other counties, it remains a relatively new and evolving field. In the USA, the Institute of Medicine has stipulated person-centred care as one of the six attributes of quality healthcare provision [15], and in the UK, there is a drive towards creating a person-centred National Health Service [16]. The World Health Organization also made a commitment to work with partners to support the implementation of people-centred healthcare [17]. However, achieving a global move to truly person-centred care will require an international paradigm shift in healthcare. Firmly embedding this concept requires a cultural shift in thinking at every level from government policy, to frontline delivery and patient expectations, facilitated by appropriate support and training [18, 19]. However, a person-centred approach should not be limited to healthcare policy and delivery but every part of the system (a whole systems approach [20]), with good public and patient involvement and coproduction at the heart of developing meaningful research and services [21].

## Why Is Person-Centred Care so Important for People Living with Obesity?

Fundamentally, patients living with obesity want and need care that is tailored to them [22–24]. The Health Foundation provides four key principles of person-centred care: providing care that is coordinated, personalised, enabling and delivered with dignity, compassion and respect [13], each of which has particular resonance for the care of people living with obesity.

### Personalised and Coordinated Care

Personalisation is at the heart of patient-centred care [25, 26]. We simply cannot just treat people as a set of diagnoses or symptoms without considering the wider social, cultural, emotional and environmental factors that impact their condition and care planning. This approach is fundamental to people living with obesity given the highly complex aetiology of the disease and multitude of associated comorbidities. It is also important to recognise person-centred care as a multidisciplinary model of care, acknowledging that some people may need support from a range of different professionals. There will also be changes and challenges to delivering person-centred care in different settings and across the life course [27]. It is therefore critical that this support is well coordinated, with systems in place to ensure prompt and effective communication between professionals, joined up care pathways, appropriate diagnostic tools and continuous patient monitoring, feedback and support [19]. Yet, despite a pledge in the NHS long-term plan [28] to provide more personalised, timely, joined up care, UK obesity patient groups frequently report (internal communication) uncoordinated, paternalistic obesity care, with barriers in communication, and poor continuity of care, which can lead to ineffective care and patient disengagement [29]. This is validated by providers of care (internal communication) who struggle to provide patient-centred joined up care within inflexible commissioning specifications and fragmented care pathways.

In the UK, primary care doctors have recently been offered the opportunity to take part in a financially incentivised “enhanced service” arrangement for weight management [30]. The specification for this service refers to making an individual assessment of readiness to engage with weight management services and referring to the most appropriate provider, thus recognising that different patients may be better suited to different services. However, most services are only available to people with certain health conditions or risk factors, and some have stated rather than individually set goals. It is contended that this does not yet represent truly person-centred care, although it demonstrates a move towards it.

### Enabling Care

With an explosion in the use of, and reliance on, the Internet, there is now a huge demand for online health information and a plethora of online resources of varying credibility [31]. Google searches for obesity related terms are significant (exceeding searches for alcohol and smoking) [32], reflecting public interest in the field, and the expanse of information available on weight management. It is therefore critical to support patients in the use and interpretation of accessible and appropriate information about their healthcare, to improve health literacy and facilitate effective joint decision making. It should however be acknowledged that different patient and professional expectations and goals may be encountered (for example, patient-centred goals may not be related to weight, i.e. non-scale victories, such as the ability to play football, or goals related to the determinants of weight, such as sleep quality or emotional wellbeing) [33, 34]. Both patient and practitioner goals and expectations are equally as important in enabling care, and this should be considered when commissioning services and setting key performance indicators.

Digital is not just impacting health literacy but also healthcare delivery, following a huge surge in telemedicine and digital health following the COVID-19 pandemic [35]. Whilst the advances in technologies such as the Internet of Things, biosensors and artificial intelligence provide important opportunities to further empower patients in self-management [36] and facilitate data-driven personalisation of care, there remains an ongoing need to optimise usability, accessibility and trust in such advances [37] in order to maximise their use and impact.

### Providing Person-centred Care with Dignity, Compassion and Respect

Ensuring person-centred care for people living with obesity is delivered with dignity, compassion and respect is critical given the alarming volume of evidence, demonstrating stigmatisation and negative treatment from healthcare providers [38•, 39, 40]. This is coupled to comprehensive evidence documenting the psychological and physiological damage associated weight stigma, which includes an increased risk of developing diabetes, disordered eating, depression, anxiety and body dissatisfaction, alongside increases in cortisol, C-reactive protein and oxidative stress [41]. Therefore, assessing weight bias using tools such as the Harvard implicit weight bias test^1^ and providing effective and compassionate communication are critical. Yet many health professionals are not confident in effectively communicating with patients about weight management, due to a lack of suitable training and knowledge [42•]. However, international consensus on the language between people living with obesity and healthcare professionals sets out the key principles of being positive, helpful and supportive; aware of non-verbal communication; collaborative; understanding; and environmentally aware. Ensuring conversations are only sought after seeking permission to initiate, always using person-first language that is free from judgement, and providing support that is evidence based and avoids humour, combat, blame, generalisation and assumptions are also essential [42•].

## The Canadian 5As: an International Framework for Person-Centred Obesity Care

In partnership with community partners and people with a lived experience of obesity, the 5As Team Research Program (5AsT) has developed a collaborative, personalised approach to address the complexity and chronic nature of obesity management [43–45]. The program aims to improve obesity prevention and management in primary care. Central to the approach are the 5As of Obesity Management™, Ask, Assess, Advise, Agree and Assist, supported by a suite of evidence based resources and tools [46••].

With a whole person lens that understands the complexity, the 5AsT approach improves individual health by integrating medical concerns with life context. It builds on people’s capacity and resilience to foster activation towards health as individuals, within their families and communities. Exploration of the syndemic effects [10] of immigrant and refugee context and the impacts of the COVID-19 pandemic are also providing further, richer understanding to extend this work, to better serve ethnically diverse, vulnerable patients with practical strategies to address social determinants of health through more seamless linkage of primary healthcare to community resources. Supporting the 5AsT approach is an international coalition of educators, clinicians, researchers, policy makers and people with lived experience who are working in partnership to support the expansion and implementation of the approach internationally. Given the international differences in healthcare systems and economies, there will be country-specific considerations and adaptations that will need to be evaluated. However, the 5AsT toolkit has been downloaded over 347 times across 33 countries around the world and has been translated into French, Spanish, German and Chinese, with Portuguese, Arabic and Swedish translations in progress. There are also plans in place to adapt and feasibility test the toolkit in the UK and an evaluation of the toolkit is currently underway in Guangzhou, China.

Introducing the 5AsT model into practice in China is complicated by differences in healthcare systems and culture. Obesity treatment does not have an obvious “home” in primary or secondary care, though the pilot study is currently in secondary care. There is also a culture of more hierarchical doctor–patient relationships compared to Canada and a preference for medications and surgery over behaviour-changing interventions, in part due to the health services and system for reimbursement. Therefore, a model of care based on supporting patients to make collaborative decisions on care is a major change, and it is uncertain if it will be successful. Evaluation of implementation is also proving challenging as patients and nurses delivering care are reluctant to give critical feedback to researchers.

Translation of language alone is not enough when transferring person-centred care frameworks from one region to another. Cultural adaptation is “the systematic modification of an evidence-based treatment (or intervention protocol) to consider language, culture, and context in such a way that it is compatible with the client’s cultural patterns, meaning, and values” [47]. This is an iterative process which starts with understanding the experiences of those living with obesity in the place where the adaptation will be implemented; this could either be from existing literature and/or specific qualitative studies. This is then followed by translation and backwards translation and adaptation of any storylines and illustrations, a process which should involve those who would be delivering and receiving the intervention. In obesity interventions, this may involve changing dietary, physical activity and health service information, and the clarity, understanding and practicality of each section should be checked with a number of stakeholders[48]. The effectiveness of the intervention should then be established within the new setting.

## Moving Forward to Global Delivery of Person-centred Obesity Care

Given the benefits of person-centred obesity care, the question remains as to why this approach is not already firmly embedded within routine practice internationally. The answer perhaps lays in the complexity of obesity and [1] how this is managed across the diversity of international healthcare systems and economies (as illustrated above), and [2] the tension between the simplicity of delivering a one-size-fits-all approach versus the complexity of delivering more personalised care. However, as the one-size-fits-all approach is not impacting global obesity prevalence, there is a need for person-centred care that follows a complex systems approach [49]. Such an approach provides a dynamic way of working to respond to complexity by bringing multidisciplinary stakeholders (including patients) to co-develop a shared understanding of the problem and a plan of action. Integral to this is the integration of system modelling, which uses big data to help understand how the system/s work, which together provides the information/evidence to provide more holistic, personalised, joined up care and a powerful framework for healthcare redesign [50].

Big data is critical in supporting a complex system approach, and therefore, more needs to be done to leverage this benefit [51]. Yet, there remains a significant gap in objective data and insights into weight management experiences, practices and outcomes across broad and diverse populations globally, which impacts the development of more personalised care. Indeed, a great deal remains to be learnt about the dynamics of behaviour change during the course of weight management journeys. There is consequently a need to combine data-driven science with people-driven discovery (systems thinking) to gain real-world insights into obesity management, to better understand what works and doesn’t work, for whom, in what context, with what resources and why.

Developing a registry of lived experiences from people living with obesity will help provide much needed evidence to improve current interventions/services and greatly inform the development of person-centred obesity care. An exciting development in this context is the expansion of the national weight control registry into the International Weight Control Registry [52], which is collecting data on the weight management initiation, weight loss and weight loss maintenance and regain. The ambition is to make this validated data widely available, accessible and usable, in order to provide a centralised authoritative evidence base to empower patients and inform the development of more tailored person-centred obesity care across the globe.

In addition to supporting healthcare redesign, advancing people-driven research and advocating policy change as we move to an era of more person-centred care, there is the need to provide more training and resources to support local implementation, delivery and evaluation. This should build upon existing resources such as those provided in the 5AsT toolkit and could include the delivery of Massive Online Open Courses to train health and care professionals across the globe. This should also be undertaken alongside the development, promotion and use of patient-reported measures to monitor the delivery and impact of person-centred obesity care [53], as it becomes an internationally embedded approach.

## Footnotes

1 https://implicit.harvard.edu/implicit/takeatest.html
